# Selection of RNAs for Constructing “Lighting-UP” Biomolecular Switches in Response to Specific Small Molecules

**DOI:** 10.1371/journal.pone.0060222

**Published:** 2013-03-26

**Authors:** Tamaki Endoh, Naoki Sugimoto

**Affiliations:** 1 Frontier Institute for Biomolecular Engineering Research (FIBER), Konan University, Kobe, Japan; 2 Faculty of Frontiers of Innovative Research in Science and Technology (FIRST), Konan University, Kobe, Japan; Argonne National Laboratory, United States of America

## Abstract

RNA and protein are potential molecules that can be used to construct functional nanobiomaterials. Recent findings on riboswitches emphasize on the dominative function of RNAs in regulating protein functions through allosteric interactions between RNA and protein. In this study, we demonstrate a simple strategy to obtain RNAs that have a switching ability with respect to protein function in response to specific target molecules. RNA aptamers specific for small ligands and a trans-activation-responsive (TAR)-RNA were connected by random RNA sequences. RNAs that were allosterically bound to a trans-activator of transcription (Tat)-peptide in response to ligands were selected by repeated negative and positive selection in the absence and presence of the ligands, respectively. The selected RNAs interacted with artificially engineered *Renilla* Luciferase, in which the Tat-peptide was inserted within the Luciferase, in the presence of the specific ligand and triggered the “Lighting-UP” switch of the engineered Luciferase.

## Introduction

Various biological functions are accomplished through allosterically-regulated interactions between biomolecules. Proteins are assumed to take the initiative in allosteric interactions with their targets, such as interactions with other proteins or the promoter regions of genomic DNAs, through structural changes in response to ligand binding or chemical modification. However, recent studies have revealed that RNA molecules also change structures to control allosteric interactions with proteins [1]–[4].

Riboswitches are RNA elements located in the untranslated region of mRNA, which can regulate gene expression through RNA conformational transition in response to specific metabolites [5]–[7]. Previous studies have identified riboswitches that regulate transcription, translation, and post-transcriptional processing in response to various types of metabolites [6], [8]. In most cases, RNA conformational transition is mediated by binding of a metabolite to an aptamer domain within the riboswitch. This binding results in the control of gene expression. The mechanism is considered simply to be completed only within RNA and its target metabolites. With regard to protein contribution, protein functions are allosterically regulated by the RNA conformational transition in some riboswitches. For example, for riboswitches that affect translational initiation, binding of the metabolite results in RNA conformational transition that allows or represses the binding of the ribosome to mRNA [9], [10]. Essentially, the molecular recognition of a specific target molecule by the aptamer domain and the subsequent RNA conformational transition results in a simple output signal. Presumably, these signals can also be produced when proteins interact allosterically with RNA. This characteristic mechanism of natural riboswitches has been mimicked to design artificial nucleic acids that enable allosteric regulation of proteins and biological functions [11].

**Figure 1:**
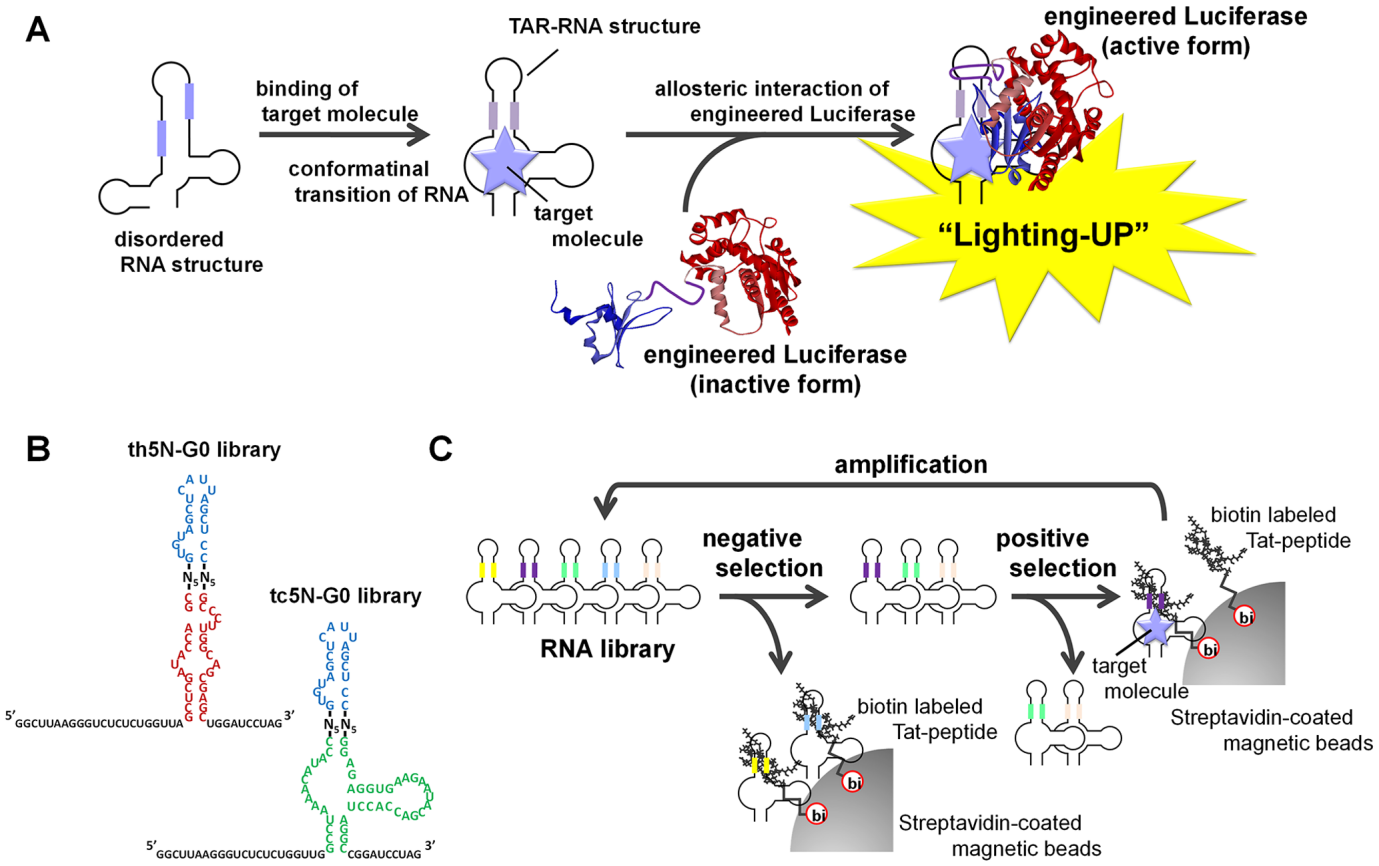
Selection of RNA for construction of the Lighting-UP switch. (A) Principle of the Lighting-UP switch on the basis of allosteric interaction between an engineered Luciferase and RNA containing a TAR-RNA and aptamer. (B) Sequences of the th5N-G0 and tc5N-G0 libraries for theophylline-responsive and tetracycline-responsive switches, respectively. Theophylline-specific aptamer, tetracycline-specific aptamer and TAR-RNA are indicated in red, green and blue, respectively. Sequences in the 5′ and 3′ termini were added as primer binding sites for amplification of RNAs by RT-PCR. N_5_ indicates a 5-nt long linker region with a random sequence. (C) Schematics showing the procedure to select RNAs that allosterically interact with the Tat-peptide in response to a target molecule.

As described previously, we constructed an artificially engineered Luciferases, in which the trans-activator of transcription (Tat)-peptide derived from bovine immunodeficiency virus (BIV) was inserted into the firefly or *Renilla* Luciferase [12], [13]. Although these engineered Luciferases considerably lost their catalytic activity because of insertion of the exogenous peptide, the activities were increased after the inserted Tat-peptide interacted with the trans-activation-responsive (TAR)-RNA, which is also derived from BIV. Presumably, the TAR-RNA induced complementation of the N and C terminal domains of the Luciferases by forcing the Tat-peptide into a β-sheet like conformation. In addition, in the case of the engineered *Renilla* Luciferase, the catalytic activity was restored to approximately 20% of the wild-type Luciferase activity and enabled intracellular detection of RNAs [12], [14]. These results led us to design “Lighting-UP” switches mediated by the allosteric interaction between the engineered Luciferase and RNA that has undergone a conformational transition to form a TAR-RNA structure in response to specific target molecules (Figure 1A).

In this study, we demonstrate the strategy for constructing the Lighting-UP switches with a selection of RNAs that allosterically interact with the BIV Tat-peptide in the presence of specific target molecules. RNA libraries containing a randomized linker sequence between the aptamers known to bind certain ligands and the TAR-RNA sequence were synthesized and selected for their ability to bind the Tat-peptide only in the presence of a target molecule. This would be a simple strategy for constructing luminometric biosensors for various target molecules because aptamers specific for various targets have already been selected. In addition, the selection was performed only for the short linker sequences.

## Materials and Methods

### Preparation of RNA libraries

th5N-G0 and tc5N-G0 libraries were prepared by *in vitro* transcription using ScriptMAX Thermo T7 Transcription Kit (Toyobo) and purified with a denaturing polyacrylamide gel. The th5N-temp DNA template had the sequence 5′-CTAGGATCCAGCTCGCTGCCAAGGGCNNNNNGGAGCTAATGA GCTACACNNNNNGCTGGTATCGAGCTAACCAGAGAGACCCTTAAGCC-3′, and the tc5N-temp was 5′-GGATCCGGC CTAGGTGGTCGT ATTCTTCACCTCTCCNNNNNGGAGCTAATGAGCTACACNNNNNGGTATGTTTTAGGCCAACCAGAGAGACCCTTAAGC-3′. th5N-temp was amplified using the 5N-PCR primer (5′-CTTAATACGACTCACTA TAGGCTTAAGGGTCTCTCTGGTT-3′) and the th5N-RT primer (5′-C TAGGATCCAGCTCGCTGCC-3′). tc5N-temp was amplified using the 5N-PCR primer and the tc5N-RT primer (5′-CTAGGATCCGGCCTAGGTGGT-3′).

### Negative selection of RNA libraries

RNA library (20 nM) was mixed with biotin-labeled Tat-peptide (100 nM), which was purchased from Scrum Inc., in a selection buffer (20 mM phosphate (pH 7.4), 100 mM NaCl, 1 mM MgCl_2_, 20 ng/ µL tRNA, 50 ng/ µL glycogen, and 0.001% (v/v) Tween 20) at final volume of 200 µL and was incubated at 37°C for 30 min. Before addition of biotin-labeled Tat-peptide, the RNA library was refolded by incubating at 70°C for 10 min and cooling to 37°C at 1°C min^−1^. Streptavidin-coated magnetic beads (Dynabeads M-280 Streptavidin: Invitrogen) (25 µL bed volume) initialized by the selection buffer was added to the reaction mixture and incubated at 37°C for 30 min. RNAs that did not bind to the Tat-peptide in the absence of trigger molecules remained in the supernatant and were ethanol precipitated for use in positive selection.

### Positive selection of RNA libraries

RNAs remaining after the negative selection were dissolved in the selection buffer (final volume of 200 µL) containing the trigger molecules (3 mM theophylline for th5N library and 100 µM tetracycline for tc5N library). The RNA library was refolded by incubating at 70°C for 10 min and cooling to 37°C at 1°C min^−1^. Biotin-labeled Tat-peptide (final concentration of 10 nM) was added and the solution was incubated at 37°C for 30 min. Streptavidin-coated magnetic beads (2.5 µL bed volume) initialized by the selection buffer was added and samples were incubated at 37°C for 30 min. The supernatant was removed, and the magnetic beads were washed 3 times in selection buffer containing the trigger molecule. RNAs were eluted from the beads in 1 M NaCl solution and ethanol precipitated.

### Amplification of RNA libraries

After the negative and positive selection, complementary DNAs of th5N and tc5N libraries were synthesized by reverse transcriptase (Rever Tra Ace: Toyobo) using th5N-RT and tc5N-RT primers, respectively. Double-stranded DNA templates for transcription of RNA libraries were synthesized by PCR using 5N-PCR primer and appropriate RT-primer, and RNAs were transcribed *in vitro* using ScriptMAX Thermo T7 Transcription Kit.

### Fluorometric analysis of interaction between RNA libraries and Tat-peptide

Tetramethyl-rhodamine (TAMRA)-labelled BIV Tat-peptide (TMR-Tat) (2.5 nM) was mixed with varying concentrations of RNA libraries in a buffer containing 20 mM phosphate (pH 7.4), 100 mM NaCl, 1 mM MgCl_2_, 20 ng/ µL tRNA, and 0.005% (v/v) Tween 20 in the absence or presence of the trigger molecules. Fluorescence intensities were measured at 37°C using a microwell plate reader (Genios Pro; Tecan) at 535 nm excitation and 590 nm emission after 60-min incubation at 37°C.

### Evaluation of equilibrium constants

The observed association constants (*K*_obs_) between the TMR-Tat and RNA libraries were calculated from the fluorescence increase as a function of RNA library concentration by statistically fitting an equation assuming an equilibrium of a simple 1∶1 binding reaction [15]. The equation follows:



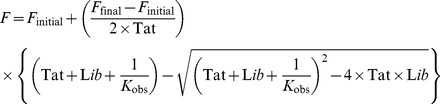
where *F* is the fluorescence signal of the TMR-Tat at each concentration of the RNA library, *F*_initial_ is the initial fluorescence signal of the TMR-Tat without RNA, *F*_final_ is the fluorescence signal when all of the TMR-Tat bound to RNA, Tat is the total TMR-Tat concentration, and Lib is the concentration of RNA library.

### Luminometric analysis of interaction between RNA libraries and engineered *Renilla* Luciferase

Plasmid vector for *in vitro* translation was constructed by transferring the coding sequence of the engineered *Renilla* Luciferase [12] to the pGSH vector, which was kindly provided from Prof. Ohtsuki [16]. The engineered *Renilla* Luciferase was translated using *in vitro* translation system (TnT Coupled Reticulocyte Lysate System; Promega). A 0.04 µL aliquot of the translated product was mixed with varying concentrations of RNA libraries and trigger molecules in a buffer containing 20 mM phosphate (pH 7.4), 100 mM NaCl, 1 mM MgCl_2_, 100 ng/ µL tRNA, and 0.005% (v/v) Tween 20 in a total reaction volume of 100 µL. The reaction mixture was incubated at 37°C for 30 min, and luminescence signals of the reaction mixture were evaluated using a multispectro microwell plate reader (Varioskan Flash; Thermo Scientific). An equal volume of 10 µM coelenterazine (Promega) diluted in reaction mixture buffer was added prior to data collection.

## Results and Discussion

### Design of RNA libraries and selection procedure

To create Lighting-UP switches that accommodate various target molecules, artificial aptamers selected by the synthetic evolution of ligands by exponential enrichment (SELEX) [17], [18] were utilized. SELEX technology has been used to select aptamers that specifically bind to proteins, metabolites including antibiotics, and small molecules, including artificial medicines [19]. Insertion of linker sequences between aptamers and functional RNAs have been successful in providing allosteric properties to functional RNAs [20]–[24]. Here, we used the linker strategy to construct the Lighting-UP switches.

As shown in Figure 1B, aptamers specific for theophylline [25], [26] and tetracycline [27] as model target molecules were connected to the TAR-RNA. The selection scheme is shown in Figure 1C. First, the RNA library was mixed with biotin-labeled Tat-peptide, and RNAs that bound to the Tat-peptide in the absence of the trigger molecule were excluded by using streptavidin-coated magnetic beads (Figure 1C; negative selection). Next, the unbound RNAs were mixed with biotin-labeled Tat-peptide in the presence of excess target molecules, and RNAs that bound to the Tat-peptide in the presence of the target molecule were separated from those that did not bind by using streptavidin-coated magnetic beads (Figure 1C; positive selection). These RNAs were amplified by RT-PCR and transcribed to prepare an RNA library for the next round of selection (Figure 1C; amplification).

The selected RNAs were not expected to adopt the TAR-RNA structure in the absence of the target molecule. The target molecule should induce an allosteric conformational transition allowing RNAs to interact with the Tat-peptide. The interaction between the TAR-RNA and the Tat-peptide is one of the strongest known RNA–peptide interactions with a dissociation constant in the low nanomolar range (−Δ*G*_25_ is approximately 11–12 kcal mol^−1^) [28], [29]. The RNA should form a disordered structure to prevent an interaction of the engineered *Renilla* Luciferase in the absence of the target molecules. Thus, the random linker sequences on either side of the TAR-RNA region were designed to be 5 nucleotides in length because the −Δ*G*_25_ of the RNA duplex is predicted to be greater than 12 kcal mol^−1^ if the random regions hybridized with regions neighboring to the opposed random regions and formed a 10-mer RNA duplex [30], [31]. In addition, the diversity of the 10-bases random sequence (1.05×10^6^) can be easily covered in a small reaction volume.

### Selection of RNA libraries

**Figure 2:**
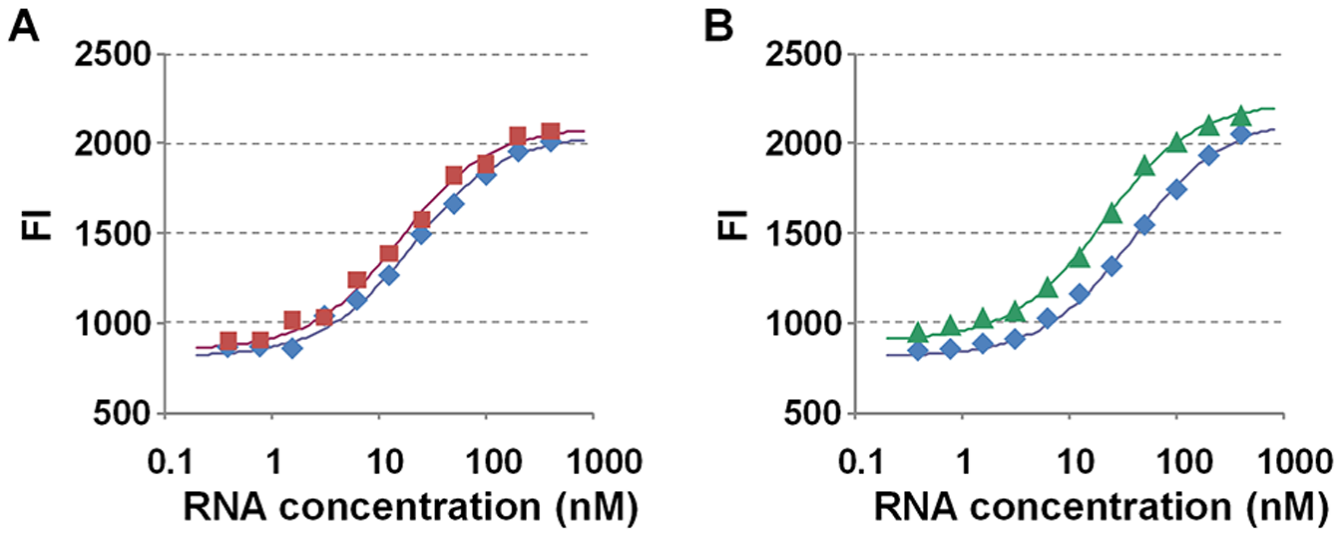
The interaction of initial RNA libraries and the Tat-peptide. (A, B) Fluorescence intensities (FI) of the TMR-Tat at 590 nm mixed with G0 RNA libraries in a buffer containing 20 mM phosphate (pH 7.4), 100 mM NaCl, 1 mM MgCl_2_, 20 ng/ µL tRNA, and 0.005% (v/v) Tween 20 at 37°C. TMR-Tat was mixed with varying concentrations of (A) th5N-G0 and (B) tc5N-G0 in the absence (blue) or presence of 3 mM theophylline (red) or 100 µM tetracycline (green).

The interaction of the initial RNA libraries for theophylline-responsive and tetracycline-responsive Lighting-UP switches (th5N-G0 and tc5N-G0 library, respectively) with the Tat-peptide was evaluated on the basis of the signal caused by binding of the TMR-Tat [32]. Libraries were mixed with the TMR-Tat in the presence and absence of the target molecules, and fluorescence signals were measured at 37°C at 535 nm excitation and 590 nm emission (Figure 2). The observed association constants for the RNA libraries and the TMR-Tat at 37°C (*K*_obs 37_) were calculated from the fluorescence signals using the equation in the Experimental Section (Table 1). The *K*_obs 37_ of the th5N-G0 and tc5N-G0 libraries in the absence of the target molecules were 0.53×10^8^ M^−1^ and 0.26×10^8^ M^−1^, respectively. Both the *K*_obs 37_ were lower than those found between wild-type TAR-RNA, whose value was 1.89×10^8^ M^−1^ in the absence of the trigger molecule (Figure S1). Thus, it was suggested that a part of RNAs in the G0 libraries did not form the TAR-RNA structure and disturbed the interaction. In the presence of 3 mM theophylline and 100 µM tetracycline, the *K*_obs 37_ for th5N-G0 (0.69×10^8^ M^−1^) and tc5N-G0 (0.51×10^8^ M^−1^), respectively, were slightly higher compared with those in their absence, although the ratios of *K*_obs 37_ in the presence and absence of the trigger molecules were less than double (1.30 and 1.96 for th5N-G0 and tc5N-G0, respectively). The results indicated that some RNAs in the G0 libraries allosterically interacted with the Tat-peptide in response to the target molecule because the *K*_obs 37_ of the wild-type TAR-RNA was almost the same regardless of the presence of the target molecules (Figure S1).

**Table 1:** Observed association constants between RNA libraries and TMR-Tat at 37°C.

	*K*_obs 37_/×10^8^ M^−1^	
RNA library	without trigger molecule	with trigger molecule
		3 mM theophylline
th5N-G0	0.53±0.07	0.69±0.10
th5N-G6	0.22±0.04	2.53±0.28
		100 µM tetracycline
tc5N-G0	0.26±0.03	0.51±0.06
tc5N-G6	0.14±0.01	1.46±0.11

**Figure 3:**
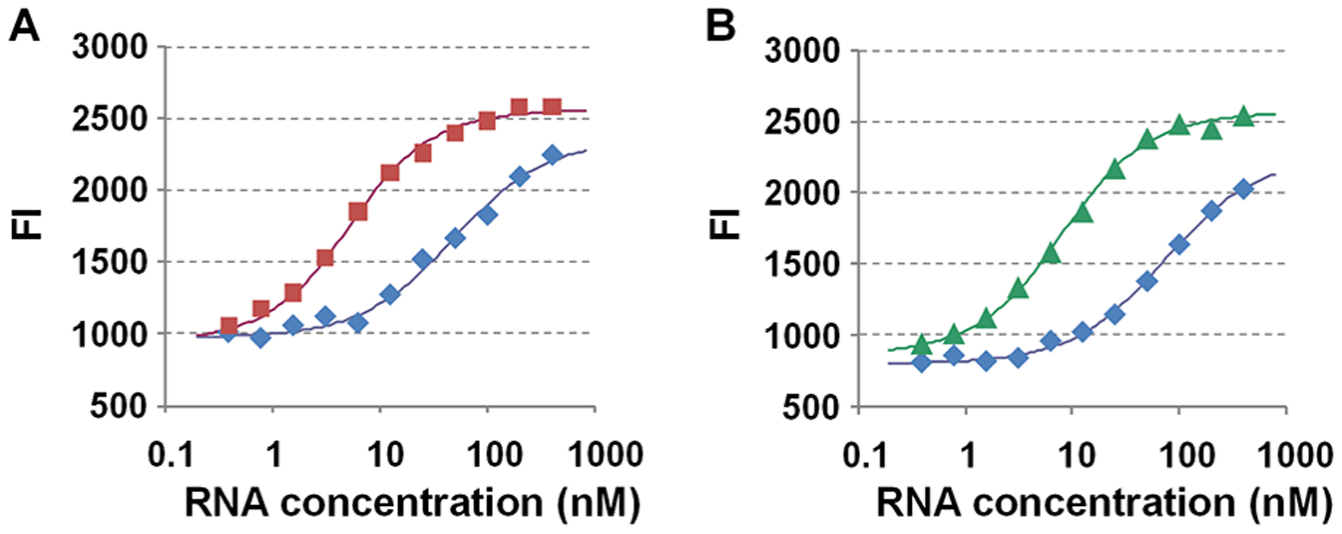
The interaction of selected RNA libraries and the Tat-peptide. (A, B) Fluorescence intensities (FI) of the TMR-Tat at 590 nm mixed with G6 RNA libraries at 37°C. TMR-Tat was mixed with varying concentrations of (A) th5N-G6 and (B) tc5N-G6 in the absence (blue) or presence of 3 mM theophylline (red) or 100 µM tetracycline (green). Buffer conditions were as described in Figure 2.

Negative and positive selections were repeated in the absence and presence of the target molecules (see Experimental Section for more detail). The results of binding of the TMR-Tat to the th5N-G6 and tc5N-G6 libraries obtained after six rounds of selection are shown in Figure 3 and Table 1. In contrast to the G0 libraries, both the th5N-G6 and tc5N-G6 libraries responded to target molecules (theophylline and tetracycline, respectively) with enhanced binding to the TMR-Tat. The *K*_obs 37_ of th5N-G6 and tc5N-G6 in the presence of the target molecules were 2.53×10^8^ M^−1^ and 1.46×10^8^ M^−1^, respectively. They were significantly higher than those of the G0 libraries and almost the same as that of the wild-type TAR-RNA. In contrast, the K_obs 37_ of th5N-G6 and tc5N-G6 in the absence of the target molecules were 0.22×10^8^ M^−1^ and 0.14×10^8^ M^−1^, respectively. They were slightly lower than those of the G0 libraries. These results indicated successful enrichment of RNAs that allosterically interact with the Tat-peptide. Our strategy first eliminated RNAs that interacted with the Tat-peptide by negative selection; only those RNAs that interacted with the Tat-peptide in response to the target molecules were subsequently selected by positive selection. The ratios of *K*_obs 37_ in the presence and absence of the specific target molecules were 11.7 and 10.1 for th5N-G6 and tc5N-G6, respectively.

### Detection of target molecules by Lighting-UP switch

**Figure 4:**
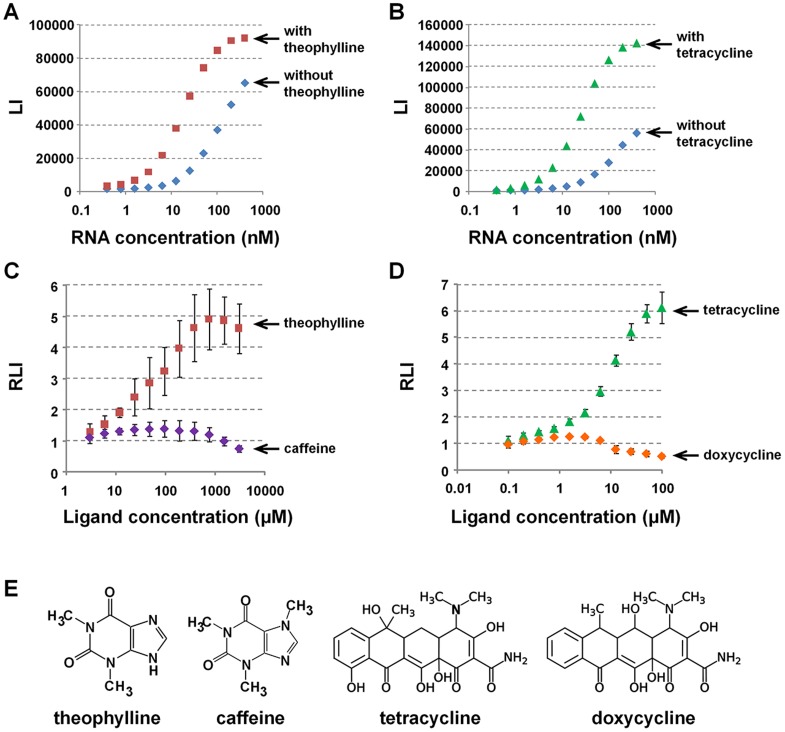
Detection of target molecules by the Lighting-UP switch. (A, B) Relative luminescence intensities (LI) of the engineered Luciferase in buffer containing 20 mM phosphate (pH 7.4), 100 mM NaCl, 1 mM MgCl_2_, 100 ng/ µL tRNA, and 0.005% Tween 20 at 37°C. An aliquot (0.04 µL) of the *in vitro* translation product was mixed with varying concentrations of (A) th5N-G6 or (B) tc5N-G6 in the absence (blue) or presence of 3 mM theophylline (red) or 100 µM tetracycline (green). tRNA was added at 100 ng/ µL to improve the signal to noise ratio. (C) Theophylline (red) or caffeine (purple) at the indicated concentrations was mixed with the *in vitro* translation product in the presence of 25 nM th5N-G6. (D) Tetracycline (green) or doxycycline (orange) at indicated concentrations was mixed with the *in vitro* translation product in the presence of 50 nM tc5N-G6. (E) Chemical structures of target molecules and analogs.

The selected RNAs were evaluated for their ability to switch the Lighting-UP of the engineered *Renilla* Luciferase in response to the target molecules. The engineered *Renilla* Luciferase was prepared by *in vitro* translation, and 0.04 µL of the crude lysate was mixed with th5N-G6 or tc5N-G6 in the presence or absence of the target molecules. The luminescence signals from the engineered *Renilla* Luciferase were analyzed at 37°C (Figures 4A and 4B). The results suggest allosteric interaction between G6 libraries and the engineered *Renilla* Luciferase in response to the target molecules because the luminescence signals increased at lower concentrations of the RNA libraries in the presence of the target molecules compared with the absence as well as the fluorescence signals in Figure 3. These results suggest that theophylline and tetracycline trigger the Lighting-UP switch of the engineered *Renilla* Luciferase at appropriate concentrations of the RNA libraries.

The concentration of th5N-G6 and tc5N-G6 were fixed at 25 nM and 50 nM, respectively, because efficient switching of the luminescence signals was expected at these concentrations and mixed with the lysate in the presence of varying concentrations of the target molecules. The luminescence signal increased depending on the concentration of the specific target molecules (Figure 4C and 4D). In addition, there were no changes in luminescence intensities in the presence of caffeine and doxycycline, analogs of theophylline and tetracycline, respectively (Figure 4E). This indicated that the aptamer moiety in the RNA libraries specifically recognized the target molecules. The specific target molecules were expected to mediate an allosteric interaction between RNA and the engineered *Renilla* Luciferase by inducing RNA conformational transition after binding to the aptamer moiety, similar to the mechanisms of natural riboswitches (Figure 1A). The switching efficiencies were calculated as the ratio of luminescence in the absence and presence of the target molecules. These were approximately 5 and 6 for th5N-G6 and tc5N-G6, respectively. We have previously performed direct detection of TAR-RNA inside cells by luminescence signal of the engineered *Renilla* Luciferase even if relative signal change was less than twice [14]. Therefore, the Lighting-UP switch constructed in this study should function as a biosensor for the intracellular detection of specific target molecules.

## Conclusions

In this study, we constructed “Lighting-UP” switches on the basis of allosteric interaction of selected RNA libraries and the engineered *Renilla* Luciferase. To prepare the RNA libraries, RNA aptamers and BIV TAR-RNA were connected via random sequences of lengths designed to preclude the formation of a TAR-RNA structure. RNAs that allosterically interacted with the Tat-peptide were enriched by repeated negative and positive selections. Although the RNA sequences were considered to have some diversity, the values of *K*_obs 37_ increased more than 10-fold in response to their specific target molecules after the sixth round of selection. The G6 libraries enabled luminometric detection of the target molecules when mixed with the engineered *Renilla* Luciferase. The target molecules, theophylline and tetracycline, were considered to induce RNA conformational change to form natural TAR-RNA conformation and the subsequent interaction of the engineered *Renilla* Luciferase that results in the complementation of the Luciferase and reactivation of the catalytic activity. Thus the selected RNAs functioned as allosteric regulators of protein function because the binding site of the target molecules and the engineered *Renilla* Luciferase were separated. We previously demonstrated fluorescent resonance energy transfer (FRET)-based detection of theophylline using theophylline-aptamer conjugated RRE-RNA and fluorescent protein consisting of Rev-peptide inserted between EYFP and ECFP [33]. Although the detection strategy is similar to this study, the rational design of RNA sequences that allosterically interact with proteins is difficult to apply other target molecules. The selection strategy performed in this study would be an easy-to-use approach to obtain RNA conformational switches for the regulation of protein function through allosteric interactions. In addition, the signal ratios in the absence and presence of the target molecules were much larger than previous FRET detection [33]. As many aptamers bind a variety of natural and artificial molecules, our strategy can be readily adapted to other target molecules to construct luminometric biosensors.

## Supporting Information

Figure S1 **The interaction of wild-type TAR-RNA with the Tat-peptide.** (A) Sequence of wild-type TAR-RNA. (B) Fluorescence intensities (FI) of TMR-Tat at 590 nm mixed with wild-type TAR-RNA in buffer containing 20 mM phosphate (pH = 7.4), 100 mM NaCl, 1 mM MgCl_2_, 20 ng/ µL tRNA, and 0.005% (v/v) Tween 20 at 37°C. TMR-Tat was mixed with varying concentrations of wild-type TAR-RNA in the absence (blue) or presence of the target molecules, 3 mM theophylline (red) or 100 µM tetracycline (green). (C) The observed association constant (*K*_obs_) for wild-type TAR-RNA and TMR-Tat at 37°C in the absence and presence of the target molecules.
(TIF)
