# Supplementary figures and images for: Selection of RNAs for Constructing “Lighting-UP” Biomolecular Switches in Response to Specific Small Molecules
Source: PLoS One. 2013 Mar 26;8(3):e60222. doi: 10.1371/journal.pone.0060222 (PMC3608610; doi:10.1371/journal.pone.0060222)

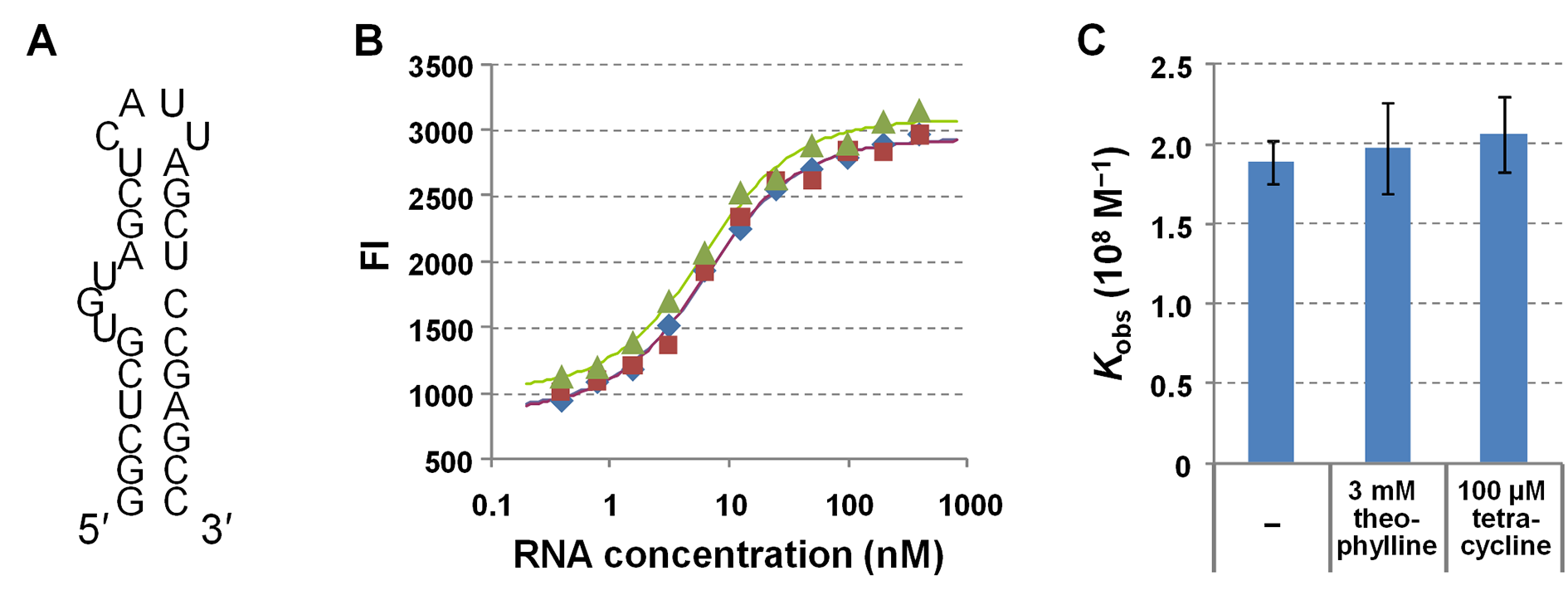

Supplement: Figure S1 — The interaction of wild-type TAR-RNA with the Tat-peptide. (A) Sequence of wild-type TAR-RNA. (B) Fluorescence intensities (FI) of TMR-Tat at 590 nm mixed with wild-type TAR-RNA in buffer containing 20 mM phosphate (pH = 7.4), 100 mM NaCl, 1 mM MgCl2, 20 ng/ µL tRNA, and 0.005% (v/v) Tween 20 at 37°C. TMR-Tat was mixed with varying concentrations of wild-type TAR-RNA in the absence (blue) or presence of the target molecules, 3 mM theophylline (red) or 100 µM tetracycline (green). (C) The observed association constant (K obs) for wild-type TAR-RNA and TMR-Tat at 37°C in the absence and presence of the target molecules. (TIF) [file pone.0060222.s001.tif]
